# Impact of Implementing Alternative Human Papillomavirus (HPV) Vaccination Strategies in Kenya: A Modeling Study

**DOI:** 10.1177/23814683261456695

**Published:** 2026-06-29

**Authors:** Valerian Mwenda, Joan-Paula Bor, Rose Jalang’o, Anne Musuva, Allison Portnoy

**Affiliations:** 1 ThinkWell, Nairobi, Kenya; 2 National Cancer Control Program, Ministry of Health, Nairobi, Kenya; 3 National Vaccines and Immunization Program, Ministry of Health, Nairobi, Kenya; 4 Department of Global Health, Boston University School of Public Health, Boston, MA, USA

**Keywords:** HPV, vaccination, cervical cancer elimination, modeling, policy, Kenya

## Abstract

**Background:**

Implementation delays reduce population-level effects of evidence-based interventions. We conducted a modeling study to estimate the effect of priority policy decisions on a human papillomavirus (HPV) vaccination program in Kenya.

**Methods:**

We used a static cohort model to estimate the health effects and costs of introduction and 1-dose switch delays, switching to nonavalent vaccine (9vHPV), supply disruptions, and various scale-up scenarios. Costs were evaluated from the health system perspective. We estimated cervical cancer cases, deaths, disability-adjusted life-years (DALYs), and program and health care costs.

**Results:**

Compared with no vaccination, maintaining current program performance would avert approximately 173,000 (95% UI: 149,000–191,000) cases and 2.7 (2.3–3.0) million DALYs across 2019 to 2100. A gradual scale-up of the current 2-dose quadrivalent vaccine (4vHPV) program would avert an additional 16,000 (14–18,000) cases (9%), increasing to 33,000 (28–37,000) cases (19%) if a multiage catchup is implemented in 2030. Accelerated scale-up of 1-dose bivalent vaccine (2vHPV) would avert 184,000 (164–199,000) cases and 3.0 (2.6–3.2) million DALYs, compared with no vaccination; translating to an additional 11,000 (2–17,000) cases averted (6%) compared with maintaining a 2-dose strategy, but at lower program ($81 million vs $76 million) and treatment costs ($2.09 billion vs $2.07 billion). HPV vaccination introduction in 2015 rather than 2019 would have averted an additional 74,000 (43%) cases. Annual vaccine supply disruptions translates to less than 9,000 (200–16,000) cases (5%) and 212,000 (74–305,000) DALYs averted (7%) compared with a 1-dose strategy. A 1-dose 9vHPV strategy would have more health benefits and save additional treatment costs compared with a 1-dose 2vHPV vaccine.

**Conclusions:**

Prompt 1-dose switch and rapid scale-up and adoption of a 9vHPV program should be priority policy decisions for Kenya.

## Graphical abstract

**Graphical abstract:**
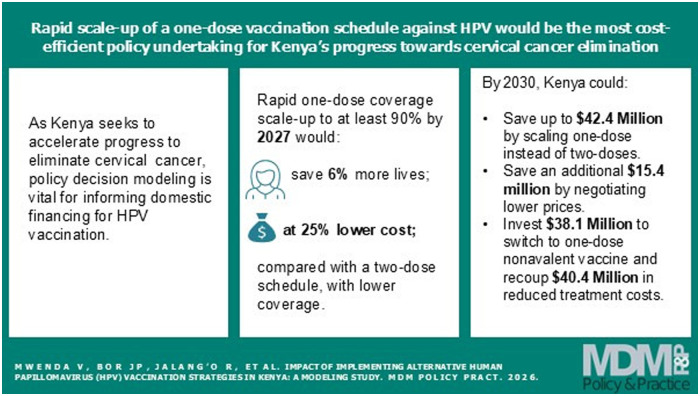

**Highlights**

- Failure to implement HPV vaccination–related policies promptly costs Kenya in terms of projected health impact.
- Implementation of a 1-dose strategy and rapid scale-up would be the most effective policy action for Kenya.
- A 1-dose nonavalent vaccine would be cost-effective compared with the gross domestic product per capita of Kenya.

## Background

The elimination of cervical cancer as a public health problem as per the global call for action by the World Health Organization (WHO) is defined as cervical cancer incidence of less than 4 cases per 100,000 female population.^1^ The scale-up of vaccination against the human papillomavirus (HPV), screening using a high-precision test (primary HPV test), and treatment of both cervical precancer and invasive cancer are estimated to reduce cervical cancer deaths by up to 89% by 2070.^2^ For this to be achieved in low- and middle-income countries (LMIC), the above interventions have to be scaled to meet WHO 2030 targets and sustained for several decades. Therefore, cervical cancer elimination will likely necessitate health system reforms that strengthen primary health care and implementation of universal health coverage.^2–5^

Population-level evidence on the effect of scaling these interventions is increasingly becoming available, and some high-income countries have elimination within their sights.^6–8^ Also, more examples of success stories on the implementation of some interventions in LMIC point to potential prospects in other settings accelerating the adoption and scaling of vaccination, screening, and treatment.^9,10^ Sub-Saharan Africa has the highest burden of cervical cancer globally, in terms of incidence and mortality,^11^ and therefore the projected impact of scaling the interventions necessary for elimination in the region could be substantial. However, in an analysis conducted in 2024 in the WHO African region, only 29 of 47 countries (62%) had introduced HPV vaccination in their national immunization programs, and only 16 (34%) had national screening programs.^12^

Due to the anticipated magnitude of investments needed by governments to scale and sustain cervical cancer prevention interventions, mathematical modeling has emerged as a relevant tool for decision making and influencing the political economy of health financing.^13^ Various health economic modeling approaches can answer relevant questions such as which policy options would be more cost-effective, what the projected costs of various implementation options would be, and what return on investment would be expected.^14–16^

Kenya has yet to attain cervical cancer elimination; the age-standardized incidence rate is 32.8 per 100,000 women,^11^ and none of the 3 prevention strategies have reached WHO elimination targets. Cervical cancer is the leading cause of cancer deaths among women in Kenya, and most of these deaths occur among women between the ages of 30 and 65 y.^17,18^ Although an HPV vaccination demonstration pilot was implemented in 2015, it was not until October 2019 that the HPV vaccine was introduced into the National Immunization Program.^19^ After introduction, the program was affected by various health system disruptions, including the COVID-19 pandemic.^20^ Despite various interventions in the last 4 y to increase vaccination coverage, it has remained consistently below 50%. Kenya has made policy-level commitments to cervical cancer elimination, primarily through the recently launched National Cervical Cancer Elimination Action Plan.^21^

As Kenya prepares to transition out of support from Gavi, the Vaccine Alliance by 2029, making an economic case for prioritization of all vaccines in the national health budget planning is paramount.^22,23^ Since transition means self-financing by countries for their immunization programs, such planning is vital for ensuring sustainability and protecting public health gains. To provide information for advocacy, previous mathematical modeling has demonstrated the cost-effectiveness of scaling up HPV vaccination coverage in the country.^24^ In addition, after the recommendation from the WHO Strategic Advisory Group of Experts on Immunization (SAGE), another modeling study focusing on a 1-dose strategy not only demonstrated improved cost-effectiveness but also identified the best use of savings resulting from the switch, further enhancing the progress toward elimination.^25^ An investment case for cervical cancer prevention and control in 2022 provided estimates for the cost of implementing elimination interventions and expected health benefits in Kenya.^26^ While these local studies have provided insights into Kenya’s cervical cancer elimination prospects, critical questions remain regarding sustainable financing for HPV vaccination, in the context of transition from donor to domestic financing.

Therefore, this study seeks to assess the impact of various policy decisions in the Kenya HPV vaccination program and make a case for adoption of approaches that could accelerate the country’s progress toward elimination.

## Methods

### Analytic Approach, Data Sources, and Assumptions

The analysis was conducted using the Harvard Scale-Up model, a static, multicohort, proportional impact model, previously described^27–29^ and internally validated against a microsimulation model of cervical carcinogenesis to ensure the validity of simplifying assumptions,^30^ which can estimate the impact of HPV vaccination on cervical cancer cases and deaths. The model estimates vaccination impact in terms of reductions in age-dependent cervical cancer incidence and mortality^17^ in direct proportion to vaccine efficacy according to vaccine type and dosing schedule, vaccine coverage, and HPV-type distribution. Age-specific population size estimates (in 1-y intervals) were sourced from the United Nations World Population Prospects 2019 revision, while age-specific life expectancy (in 5-y intervals) was obtained from 2019 World Health Organization (WHO) life tables. Country-specific cervical cancer incidence data were sourced from Globocan 2020 (International Agency for Research on Cancer; IARC); Kenya has 2 high-quality population-based cancer registries that regularly submit data to IARC.^31^ The model accounts for current and future health and economic benefits at the population level considering changing demographics (eg, population size, mortality rates) over time.

The model captures burden from all HPV genotypes, but the impact of vaccination is limited to the burden caused by genotypes targeted by the vaccine. In this analysis, the model simulated health benefits from vaccination against HPV-16/18 (the 2 oncogenic HPV types in the quadrivalent HPV vaccine [4vHPV] in Kenya’s current immunization schedule) or HPV-16/18/31/33/45/52/58 (the oncogenic HPV types in the nonavalent HPV vaccine [9vHPV]). The proportion of cervical cancer attributable to the HPV genotypes in Kenya was assumed to be 72.9% (95% uncertainty interval: 59.6%–86.2%) for HPV-16/18 and 86.9% (83.8%–89.7%) for HPV-16/18/31/33/45/52/58.^32^ The model assumed that the age-specific cervical cancer incidence among unvaccinated women remained constant over the time horizon of the model.

To estimate cancer mortality, the model first assumed Kenya-specific distributions of cancer stages for incident cancers across the time horizon of the analysis: 9% (stage I), 36% (stage II), 47% (stage III), and 8% (stage IV).^2^ The model then incorporated 5-y stage-specific survival probabilities for untreated and treated cervical cancers and treatment access proportions. These values are combined into weighted averages to provide the 5-y probability of death parameters by stage—83.6% (stage I), 85.5% (stage II), 89.1% (stage III), and 98.3% (stage IV)—and validated against age-specific mortality rates.^2,17^ In other words, incident stage-specific cancers face the relevant stage-specific probability of death over a 5-y period.

For 2-dose vaccination, we assumed 100% vaccine efficacy^33–37^; for 1-dose vaccination, we assumed single-dose nonavalent vaccine efficacy was 98.8% (91.3%–100%) and single-dose bivalent vaccine (2vHPV) efficacy was 97.5% (90.0%–100%) based on 36-mo trial data.^38,39^ We assumed that a switch to a single-dose regimen in Kenya would involve a switch from quadrivalent to bivalent vaccine, as these are the single-dose trial data available. All vaccine regimens were assumed to confer lifelong protection.

For each vaccination schedule (either 1 or 2 doses), the model assumed that females targeted for vaccination are fully immunized with perfect timeliness at the target ages and that those effectively immunized against vaccine-targeted HPV types can develop cervical cancer associated with nonvaccine HPV types. In this analysis, we did not assume any cross-protection against nonvaccine types and did not examine indirect (ie, herd) effects.

We assumed a vaccine price of $4.50 per dose for HPV vaccine, which corresponds to the subsidized cost of HPV vaccine procured by Gavi for LMIC such as Kenya. We also included costs for vaccine wastage at a rate of 5% for a single-dose vial, liquid formulation.^40^ Lastly, we assumed a 1-time introduction cost of $2.00 per girl in the first year of the vaccination program, followed by a recurrent delivery cost of $1.70 per girl, including costs for personnel, training, social mobilization, disease surveillance, program management, and other recurrent costs.^41,42^ Since Kenya is expected to transition from Gavi support by 2029, we assumed that posttransition, the country will procure vaccines at market prices and included scenarios with either higher or lower vaccine prices in the analysis.

We adjusted previously published treatment costs for Kenya to 2024 US dollars using local consumer price index and exchange rates, assuming that local cancer is equivalent to stage I cancer, regional cancer is equivalent to stage II or III cancer, and distant cancer is equivalent to stage IV cancer, resulting in $1,547.29 for stage I, $7,490.74 for stage II and stage III, and $5,925.27 for stage IV.^43^ We assumed in the base case that 90% of women with detected cervical cancer would have access to cervical cancer treatment and incur the relevant treatment costs, per the WHO cervical cancer elimination target. In a scenario analysis, we assumed that cervical cancer treatment costs applied only to the estimated proportion of women with access to radiation therapy in Kenya (assumption of 30%, based on the current structure of cancer care provision in Kenya; Appendix S2)^44^; the remainder of women incurred no costs for cancer treatment.

### Analytic Scenarios and Outcomes

We conducted analyses to evaluate the impact of HPV vaccination assuming varying vaccine delivery strategies for cohorts vaccinated in 2026 to 2030, with all strategies assuming the same historical vaccine program implementation experienced in Kenya (Appendix S1). In the base-case scenario (scenario 0), we assumed ongoing routine 2-dose vaccination coverage of 10-y-old girls at 50% and transition from Gavi support in the year 2029. Scenario 1 is similar to scenario 0 but with coverage being scaled to 90% by 2030. Scenario 2 is similar to 1 but with a multiage cohort (MAC) catch-up in 2030. Scenario 3 assumes a 2-dose strategy with gradual scale-up similar to scenario 1 but with a nonavalent (9vHPV) vaccine. Scenario 4 simulates the impact of introducing the HPV vaccine in 2015 (when Kenya conducted a pilot), to demonstrate the impact of policy implementation delays. Scenario 5 explores the likely budget impact if transition from Gavi happened earlier, in the context of a potential reduction of financing to Gavi by major financing partners. Scenario 6 explores the potential impact of supply disruptions and is based on supply disruptions witnessed in recent years.^45^ Scenarios 7 through 12 explore the impact of the country adopting single- or 2-dose strategies at different price points. The gradual scale-up assumption is based on the projected coverage in the national cervical cancer elimination plan. We examined changes to coverage, vaccine type, dosing schedule, adding multicohort vaccination campaigns, supply disruptions, vaccine price, and Gavi transition timeline. The full list of scenarios is defined in Table 1.

**Table 1.** Analytic Scenarios.

Scenario	Vaccine	Pre-2025 Vaccination Coverage	Future Vaccination Coverage	Future Dose Schedule	Age Cohort(s)	Vaccine Price	Treatment Access Level
Scenario 0: The country maintains the current strategy (2-dose 4vHPV), and average coverage remains 50% until 2030	4vHPV	Historical^a^	2026+: 50%	2-dose	Historical^a^ + 10-y-old routine annual 2026+	Until 2028: $4.50 2029+: $13.50	30%, 90%
Scenario 1: The country maintains the current strategy (2-dose 4vHPV) and there is gradual scale-up	4vHPV	Historical^a^	2026: 50% 2027: 60% 2028: 70% 2029: 80% 2030: 90%	2-dose	Historical^a^ + 10-y-old routine annual 2026+	Until 2028: $4.50 2029+: $13.50	30%, 90%
Scenario 2: The country maintains the current strategy (2-dose 4vHPV) and there is gradual scale-up and a multiage cohort (MAC) catch-up in 2030	4vHPV	Historical^a^	2026: 50% 2027: 60% 2028: 70% 2029: 80% 2030: 90%	2-dose	Historical^a^ + 10-y-old routine annual + 10- to 14-y-old MAC in 2030	Until 2028: $4.50 2029+: $13.50	30%, 90%
Scenario 3: Switch to 9vHPV but maintain 2-dose strategy	9vHPV	Historical^a^	2026: 50% 2027: 60% 2028: 70% 2029: 80% 2030: 90%	2-dose	Historical^a^ + 10-y-old routine annual 2026+	Until 2025: $4.50 2026-2028: $13.50 2029+: $33.25	30%, 90%
Scenario 4: The country introduced a 2-dose 4vHPV schedule in 2015	4vHPV	No delay (2015 introduction)	2026: 50% 2027: 60% 2028: 70% 2029: 80% 2030: 90%	2-dose	Historical^a^ + 10-y-old routine annual 2026+	Until 2028: $4.50 2029+: $13.50	30%, 90%
Scenario 5: The country maintains the current strategy (2-dose 4vHPV) and there is gradual scale-up, but it transitions out of Gavi support in 2026 not 2029	4vHPV	Historical*	2026: 50% 2027: 60% 2028: 70% 2029: 80% 2030: 90%	2-dose	Historical* + 10yo routine annual 2026+	Until 2025: $4.50 2026+: $13.50	30%, 90%
Scenario 6: The country maintains the current strategy (2-dose 4vHPV), and there is gradual scale-up, but there are annual supply disruptions affecting a quarter of expected doses.	4vHPV	Historical*	2026: 38% 2027: 45% 2028: 53% 2029: 60% 2030: 68%	2-dose	Historical* + 10yo routine annual 2026+	Until 2028: $4.50 2029+: $13.50	30%, 90%
Scenario 7: The country switches to one-dose strategy (2vHPV), and has accelerated coverage scale-up	2vHPV	Historical^a^	2026: 50% 2027–2030: 90%	1-dose	Historical^a^ + 10-y-old routine annual 2026+	Until 2028: $4.50 2029+: $13.50	30%, 90%
Scenario 8: The country switches to 1-dose strategy (2vHPV) and has accelerated coverage scale-up, but the vaccine has a higher price	2vHPV	Historical^a^	2026: 50% 2027–2030: 90%	1-dose	Historical^a^ + 10-y-old routine annual 2026+	Until 2028: $4.50 2029+: $26.75	30%, 90%
Scenario 9: The country switches to 1-dose 9vHPV, with accelerated scale-up	9vHPV	Historical^a^	2026: 50% 2027–2030: 90%	1-dose	Historical^a^ + 10-y-old routine annual 2026+	Until 2025: $4.50 2026–2028: $13.50 2029+: $33.25	30%, 90%
Scenario 10: The country maintains a 2-dose strategy, 4vHPV but with a lower vaccine price	4vHPV	Historical^a^	2026: 50% 2027: 60% 2028: 70% 2029: 80% 2030: 90%	2-dose	Historical^a^ + 10-y-old routine annual 2026+	Until 2025: $4.50 2026+: $2.90	30%, 90%
Scenario 11: The country switches to 1-dose strategy (2vHPV) and has accelerated coverage scale-up, but the vaccine has lower price	2vHPV	Historical^a^	2026: 50% 2027–2030: 90%	1-dose	Historical^a^ + 10-y-old routine annual 2026+	Until 2025: $4.50 2026+: $2.90	30%, 90%
Scenario 12: The country switches to 1-dose strategy (2vHPV) but targets a MAC until coverage reaches 90% and then SAC of 10 y	2vHPV	Historical^a^	2026: 50% 2027: 60% 2028: 70% 2029: 80% 2030: 90%	1-dose	Historical^a^ + 10-y-old routine annual + 11- to 14-y-old MAC in 2030	Until 2028: $4.50 2029+: $13.50	30%, 90%

*Note.* 2vHPV, bivalent HPV vaccine; 4vHPV, quadrivalent HPV vaccine; 9vHPV, nonavalent HPV vaccine; HPV, human papillomavirus.

a This is the actual coverage for the period 2019 to 2023.

The primary outcomes of the analysis included the lifetime costs associated with the vaccination strategies, comprising the cost of the vaccine program (including dosage and delivery costs) and the cost of cervical cancer treatment (reflecting costs incurred for treatment of cervical cancer and cost offsets due to cancer prevention following HPV vaccination) in 2024 US dollars. We estimated the potential health impact in terms of cervical cancer cases, deaths, and disability-adjusted life-years (DALYs) among vaccinated cohorts from the time of vaccination until year 2100 (compared with no vaccination). In calculating DALYs, all cervical cancer cases experienced up to 5 y lived with disability according to the following disability weight assumptions: 0.288 for the first year of stages I to III cervical cancer, 0.049 for years 2 to 4 of stages I to III cervical cancer, 0.451 for years 1 to 4 of stage IV cervical cancer, 0.172 for the first 9 mo of year 5 of stages I to III cervical cancer, 0.473 for the first 9 mo of year 5 of stage IV cervical cancer, and 0.54 for the last 3 mo in year 5 (before death) for all stages.^46,47^ We discounted both future costs and DALYs at a rate of 3% annually (ie, beginning in 2026).

To compare a single-dose 2vHPV regimen with a single-dose 9vHPV regimen, we calculated the incremental cost-effectiveness ratio (ICER), defined as the additional cost of a particular strategy divided by the additional health benefits (ie, DALYs averted), compared with the next less-costly strategy. The estimated ICERs were compared with a cost-effectiveness threshold of $1952.30 per DALY averted, the gross domestic product (GDP) per capita in Kenya in 2023,^48,49^ which represented the willingness of the health care system in Kenya to pay for care, in order to identify the optimal strategy. Use of GDP per capita has been proposed as one alternative approach for countries without a locally established cost-effectiveness threshold.^50^

### Sensitivity Analysis

Five key parameters were identified for probabilistic sensitivity analysis (PSA): HPV type distribution, age-specific cervical cancer incidence, stage distribution of cervical cancer, stage-specific 5-y survival and treatment access (as a combined parameter), and 1-dose vaccine efficacy. Each parameter was assigned a β-PERT distribution for probabilistic sampling, with the bounds determined by 1) empirical data for HPV-type distribution and vaccine efficacy,^32,38,39^ 2) confidence intervals estimated from cervical cancer cases in GLOBOCAN 2022,^17^ and 3) assumed ±10% bounds from the base case for stage distribution and stage-specific probability of death following 5-y survival, if this estimate is contained between 0 and 1.

For type distribution, bound width was determined by the regional minimum or maximum across all countries in the African region, whichever was farther away from the regional average, such that bounds are symmetric. For age-specific cervical cancer incidence, confidence intervals were derived from cervical cancer cases using a published formula.^51^ The estimated intervals were multiplied by 3 to incorporate additional uncertainty due to reliance on GLOBOCAN estimated cases rather than registry data. For stage distribution, for a single parameter set, the value for a single stage (stage II) is drawn individually, and the remaining stages are normalized to adjusted values in order for the 4 stages to add up to 1. For the stage-specific probability of death, as a probability cannot exceed 100%, the minimum bound was set to the difference between the base-case value and 1, where relevant. Two hundred independent parameter sets were drawn for PSA.

## Results

### Projected Health Impact and Cost of Alternative Policy Choices

Table 2 shows the projected health impact and cost implications of each of the scenarios considered. Compared with no vaccination, if the country continued on its current trajectory (scenario 0, 2-dose 4vHPV, with average coverage at 50%), then approximately 173,000 (95% uncertainty interval: 149,000–191,000) cervical cancer cases and 2.7 (2.3–3.0) million DALYs would be averted within the next 100 y. A gradual scale-up of vaccination coverage to 2030 (scenario 1) would avert an additional 16,000 (14–18,000) new cases, but this number would double to 33,000 (28–37,000) cases (19% more cases averted) if the country would, in addition, implement a MAC catch-up campaign in 2030 (scenario 2), to address the missed opportunities due to suboptimal coverage in the intervening period. If the country were to implement a 1-dose 2vHPV strategy (scenario 7), with accelerated scale-up as a result, then 184,000 (164–199,000) cases of cervical cancer and 3.0 (2.6–3.2) million DALYs would be averted, compared with no vaccination; this would translate to an additional 11,000 (2–17,000) cases averted (6%) compared with maintaining current status (scenario 0), but at a lower vaccination program cost ($81 million vs $76 million) as well as lower treatment costs ($2.09 billion vs $2.07 billion).

**Table 2.** Impact of Various Scale-up Options of the Current HPV Vaccination Strategies in Kenya, Assuming 90%^a^ Access to Cancer Treatment.

Implementation Scenario	Total Cases Averted	Total DALYs Averted (Thousands)	Total Deaths Averted	Years of Life Saved (Thousands)	Costs (Millions, 2024 USD)			
					Vaccine	Delivery	Total Vaccination	Treatment
Scenario 0	172,713	2,701.6	142,284	2,580.9	62.7	19.1	81.8	2,086.0
Scenario 1	188,867	2,977.6	155,302	2,845.8	77.3	21.4	98.7	2,061.1
Scenario 2	205,251	3,257.4	168,521	3,114.2	95.5	23.6	119.1	2,035.9
Scenario 3	204,391	3,217.6	168,067	3,074.9	144.7	21.4	166.1	2,037.3
Scenario 4	246,942	3,908.6	204,837	3,735.7	99.0	29.1	128.1	1,971.9
Scenario 5	188,867	2,977.6	155,302	2,845.8	99.1	21.4	120.5	2,061.1
Scenario 6	174,902	2,739.1	144,045	2,616.9	67.0	19.4	86.4	2,082.6
Scenario 7	184,035	2,951.3	150,689	2,823.1	58.7	18.0	76.7	2,068.6
Scenario 8	184,035	2,951.3	150,689	2,823.1	74.9	18.0	92.9	2,068.6
Scenario 9	210,259	3,375.5	172,111	3,229.1	96.8	18.0	114.8	2,028.2
Scenario 10	188,867	2,977.6	155,302	2,845.8	49.0	21.4	70.3	2,061.1
Scenario 11	184,035	2,951.2	150,689	2,823.1	43.3	18.0	61.3	2,068.6
Scenario 12	173,316	2759.5	141,912	2,638.7	85.3	19.6	104.9	2,085.0

a The results for the 30% treatment access assumption are provided in Appendix S2.

Based on current contextual realities (transition from Gavi, reduction in donor financing for health, and recurrent disruption to vaccine supplies), we also examined the impact of an earlier than expected transition from Gavi (scenario 5); such an eventuality would mean an additional $39 million in total program costs compared with the current coverage situation and transition as planned by 2029. Since the country has been reporting annual disruptions in vaccine supplies for the past 2 y, we made an assumption that this would decrease the overall program performance by 25% (scenario 6); the decreased impact would be approximately less 9,000 (200–16,000) cases (5% change) and 212,000 (74–305,000) DALYs averted (7% change), compared with a 1-dose strategy. If the country were to adopt a 9vHPV, 1-dose strategy (scenario 9), the impact would be 210,000 (196–221,000) cases and 3.4 (3.1–3.6) million DALYs averted compared with no vaccination, equivalent to an additional 38,000 (21–59,000) cases (22%) and 674,000 (405–1,007) DALYs (25%) averted compared with current program status (scenario 0), and additional 26,000 (12–46,000) cases (14%) and 424,000 (187–746,000) DALYs (14%) averted, compared with an accelerated coverage 1-dose 2vHPV program (scenario 7). A switch to a 1-dose schedule with a gradual increase in coverage and a MAC targeting 11- to 14-y-old girls in 2030 (scenario 12) would avert 7,200 fewer (11,800 fewer to 2,100 more) cases and 128,751 fewer (206,600 fewer to 30,600 more) DALYs compared with a switch to 1-dose alongside a rapid increase in coverage (scenario 7), but with an uncertainty interval that overlaps scenario 7 and comes at a higher cost—an additional $28.2 ($25.0–46.3) million.

### Potential Impact of Policy Implementation Delays

**Figure 1.**
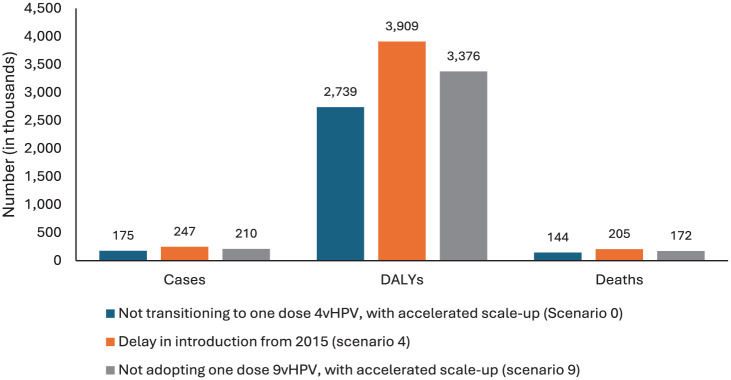
Potential program impact forgone by policy-level decisions.

Figure 1 shows the potential impact of HPV program policy implementation delays in the context of cervical cancer elimination in Kenya. If Kenya would have implemented the HPV vaccination program immediately following the pilot in 2015 (scenario 4), then the country would have averted 247,000 (215–279,000) cases and 3.9 (3.4–4.5) million DALYs in 100 y, compared with no vaccination. This would have been 1) 74,000 (43%) more cases averted compared with current vaccination status (scenario 0), 2) 58,000 (31%) more cases averted compared with a gradual scale-up from 2026 to 2030 (scenario 1), 3) 42,000 (21%) more cases averted compared with gradual scale-up and a MAC catch-up in 2030 (scenario 2), and 4) 63,000 (34%) more cases averted compared with switching to a 1-dose strategy and accelerated scale-up from 2026 (scenario 7). Not transitioning to a 1-dose regimen and accruing the program benefits from 2026 would mean forgone impact of 11,300 (7%) cases averted and 8,400 (6%) deaths averted, compared with current status (scenario 0).

### Budgetary Implication of Alternative Policy Decisions on the HPV Vaccination Program

**Figure 2.**
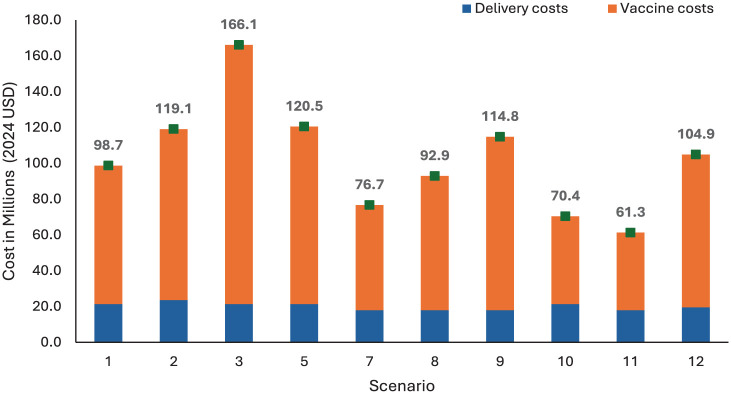
Cost of the immunization program, by scenario, Kenya. Scenario 1: 2-dose 4vHPV, gradual scale-up; scenario 2: 2-dose 4vHPV, multiage cohort (MAC) in 2030; scenario 3: switch to 2-dose 9vHPV; scenario 5: 2-dose 4vHPV, but Gavi transition in 2026; scenario 7: switch to 1-dose 2vHPV, accelerated scale-up, current vaccine price; scenario 8: switch to 1-dose 2vHPV, accelerated scale-up; higher vaccine price; scenario 9: switch to 1-dose 9vHPV, accelerated scale-up; scenario 10: 2-dose 4vHPV, but lower vaccine price; scenario 11: switch to 1-dose 2vHPV, accelerated scale-up, lower vaccine price; scenario 12: switch to 1-dose strategy (2vHPV) but targets a MAC until coverage reaches 90% and then single-age cohort of 10 y.

Figure 2 shows the undiscounted cost of health services and the vaccination program by various policy/implementation scenarios in Kenya. While program delivery costs remain relatively constant for all policy options (approximately $20 million), the lowest vaccine costs would be switching to a 1-dose 4vHPV, with a lower ($2.90) than current ($4.50) price (scenario 11). The costliest option would be switching to a 2-dose, 9vHPV strategy, assuming a higher price ($13.50) for this vaccine (scenario 3). Maintaining a 2-dose 4vHPV strategy but with lower prices (scenario 10) would cost the same as a 1-dose strategy with current prices (scenario 7) but with lower projected program coverage and impact. Kenya would save approximately $22 million by 2030 if it switches to 1 dose at current prices and up to $37 million if cheaper vaccine prices become available. Therefore, vaccine type, cost, and delivery strategy/schedule are key policy considerations in the Kenya context.

Due to policy and stakeholder interest to consider switching from 2vHPV to 9vHPV, we considered the cost-effectiveness of such a change, assuming 1-dose delivery strategies (Table 3). We found that such a change would be cost-effective compared with Kenya’s GDP per capita and provide greater health benefits.

**Table 3.** Discounted Costs (2024 USD), Disability-Adjusted Life-Years (DALYs), and Incremental Cost-Effectiveness Ratios of Potential Switch from Bivalent (2vHPV) Vaccine to Nonavalent (9vHPV) Vaccine in the Routine Female Vaccination Program in Kenya, Assuming 90% Access to Cervical Cancer Treatment.

Strategy	Total Costs (Millions)	Total DALYs Averted	Incremental Costs (Millions)	Incremental DALYs Averted	Cost per DALY Averted
Scenario 7: 2vHPV 1 dose	$647	846,605			
Scenario 9: 9vHPV 1 dose	$672	965,194	$24.9	118,579	$210

*Note.* HPV, human papillomavirus.

**Figure 3.**
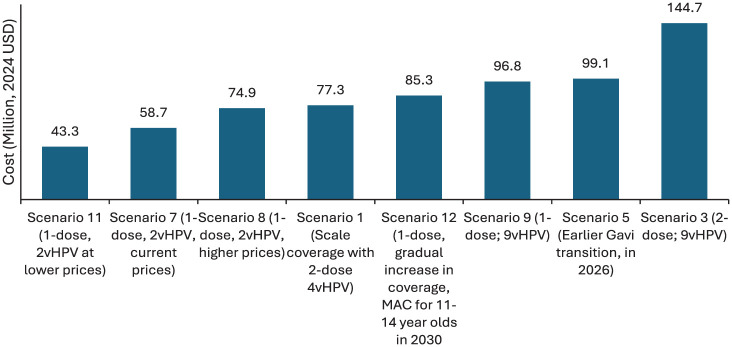
Vaccine costs based on potential policy decisions in Kenya.

Figure 3 shows the undiscounted vaccine costs depending on various policy options that Kenya can potentially pursue, from the least to the highest budget impact. The lowest cost option would be transitioning to a 1-dose 2vHPV vaccine, with lower vaccine prices than the current Gavi amount (scenario 11). The costliest options would either be switching to a 9vHPV vaccine type (also 1 dose) or an earlier than expected full transition from Gavi.

## Discussion

We found that Kenya could avert an additional an additional 16,000 (9%) cervical cancer cases if the country had a gradual scale-up of the current 2-dose 4HPV strategy to 2030 (scenario 1), compared with the coverage remaining at 50% (scenario 0). If a gradual scale-up of the current strategy was implemented and then a MAC catch-up implemented in 2030 (scenario 2), additional cases averted would double to 33,000 (19%) compared with the status quo (scenario 0). Switching to 1 dose with accelerated coverage scale-up, the 4vHPV (scenario 7) strategy would not only result in an additional 11,000 (6%) cases averted over the status quo scenario 0 (and 184,000 cases compared with no vaccination) but also at a lower cost. A 1-dose strategy with 9vHPV (scenario 9) would avert an additional 38,000 cases but at a higher vaccination cost. At a reduced price for 9vHPV, we would expect additional cost savings such that a 1-dose 9vHPV program might be cost saving compared with 1-dose 2vHPV overall. If the country maintains a 2-dose strategy and implements a gradual scale-up to 90% by 2030, but with periodic disruptions to vaccine supply as transitions progress (scenario 6), then the country would forgo a benefit of an additional 14,000 cases that would otherwise have been averted. By not transitioning to a 1-dose strategy and therefore potentially having recurrent annual supply disruptions, Kenya would fail to avert nearly 300,000 DALYs and 10,000 additional deaths from cervical cancer. With a single-dose schedule, a rapid increase in coverage is more impactful and cheaper than a gradual increase in coverage and a MAC catch-up targeting girls 11 to 14 y in 2030.

To demonstrate the impact of policy delays, we estimated the potential impact of the country not implementing the HPV vaccination immediately following the 2015 pilot (scenario 4). Kenya would have averted 74,000 (43%) more cervical cancer cases and saved $114 million in treatment costs, compared with current status (scenario 0). This represents the opportunity lost due to delay in implementation of policies. Therefore, this has implications, especially in the current delay of implementation of a 1-dose strategy, despite local health and economic evidence, as well as advisory by the National Immunization Technical Advisory Group recommendation.

To advance toward cervical cancer elimination in Kenya, the country may need to implement key policy decisions. One, prompt implementation of the 1-dose approach would not only be cost-effective but would also provide opportunities for scale-up and sustainability of high coverage. One gain, for instance, would be reduced vaccination costs as the country transitions out of Gavi support and in the context of declining overall developmental assistance globally. Second, contingency planning may be necessary. If funding for Gavi was markedly reduced, then one possible outcome may be a more abrupt decline in funding for countries already in the transition phase. For the Kenyan context, this would mean funding requirements exceeding $120 million if the country maintains a 2-dose strategy and $92 million given a transition to a 1-dose strategy but with a higher vaccine price. Third, if the country were to implement a 1-dose 9vHPV vaccine strategy, then, an additional 26,000 cases (14%) would be averted over a 1-dose 2vHPV strategy. Most importantly, an additional vaccination cost of $38 million from adopting a 9vHPV vaccine would be negated by a saving of $40 million from lower treatment costs due to greater reductions in cervical cancer in a 9vHPV versus a 2vHPV strategy. On vaccination costs, the most attractive choice for Kenya would be switching to a 1-dose 2vHPV strategy as well as considering adoption of cheaper vaccines that are increasingly becoming available. Whatever the choice, the HPV vaccination program requires substantial upfront investment, but the long-term benefits of averting cervical cancer and the associated health care and economic costs is worthwhile in the Kenyan context. Therefore, as Kenya pursues health financing transition, from donor to domestic sources, our analysis provides insights for the projected budget impact of various policy decisions in the HPV vaccination program.

Our findings are consistent with prior evidence, especially those focused on estimating the impact of policy delays like implementation of 1-dose strategies. Burger et al^52^ estimated an addition of 10% more deaths averted by HPV vaccination when comparing immediate implementation of single-dose vaccine delivery with a delay of 5 y in an exemplar high-burden setting. This implies that any amount of delay translates to a longer elimination horizon, especially for countries who have not yet achieved optimal coverage. A modeling study in China demonstrated the impact of delaying scaling of HPV vaccination, even with expansion of screening and treatment.^53^ This study by Gao et al demonstrated that a delay of 8 y in scaling HPV vaccination but rapidly scaling HPV testing would still cause a reduction in cases averted of up to 3.4% or more than 500,000 cases. In Norway, Portnoy et al^54^ estimated the impact of delayed implementation of both HPV vaccination and HPV-based screening on the elimination timeline, making a case for swift implementation once evidence for clinical effectiveness, cost-effectiveness, and health system capacity is available.

This modeling study has important limitations. First, the scenarios for switching to a 1-dose strategy were based on an optimistic coverage assumptions: 90% by 2027. If other barriers to HPV vaccination in Kenya are not addressed, then accelerated coverage may not be realized. Without this, then the adoption of a 1-dose strategy would not produce the anticipated program benefits. Second, projecting the effect of various policy decisions across all the pillars of the elimination strategy, that is, including screening and treatment, could be more informative for decision making. In particular, investments in screening and treatment would likely project an earlier population impact on cervical cancer outcomes.^2^ Such an analysis, however, would require a dynamic transmission model that would incorporate screening and treatment levers. Our model makes an assumption that screening remains unchanged. While treatment of invasive cancer is included in the model, screening and treatment of precancer is not. Currently, the uptake of screening using high-precision methods is very low (less than 10%). In the event that the country scales up screening and precancer treatment rapidly before 2030, the overall health outcomes in the cervical cancer program would be different from the scale-up of vaccination alone. Third, age-specific cervical cancer incidence, stage-specific cervical cancer mortality, and stage distribution assumptions are held constant across the time horizon of the analysis. The simulation of future outcomes comes with inherent uncertainty, but we might expect that potential future improvements in the cervical cancer screening program in Kenya could alter stage distribution, with cancer likely being detected at earlier stages and therefore decreasing cervical cancer mortality. Although the health gains from our vaccination scenarios may therefore be overestimated, the ranking between scenarios is unlikely to differ as the majority of policy changes analyzed take place over a 5-y time frame. Fourth, this being a static model, it does not incorporate the potential impact of herd (indirect) protection and therefore could be prone to biased estimates; herd effects have been shown to be a significant factor in HPV vaccine population-level effectiveness, especially where vaccination coverage is low.^55^

In conclusion, to be on the path to cervical cancer elimination, it will be important to avoid policy or programmatic delays to the implementation of evidence-based approaches at scale. Switching to a 1-dose strategy promptly and using the cost and logistical benefits to scale coverage would be the most advisable policy action at the moment. Considering a switch to a 9vHPV vaccine in the vaccination program could have greater impact on cervical cancer cases averted, without cost implications on the overall program (considering averted treatment costs). Such a decision, coupled with pursuit of cheaper vaccine alternatives, could form part of the intermediate policy options for a high-burden country such as Kenya.

## Supplemental Material

sj-docx-1-mpp-10.1177_23814683261456695 – Supplemental material for Impact of Implementing Alternative Human Papillomavirus (HPV) Vaccination Strategies in Kenya: A Modeling Study Supplemental material, sj-docx-1-mpp-10.1177_23814683261456695 for Impact of Implementing Alternative Human Papillomavirus (HPV) Vaccination Strategies in Kenya: A Modeling Study by Valerian Mwenda, Joan-Paula Bor, Rose Jalang’o, Anne Musuva and Allison Portnoy in MDM Policy & Practice
