# Supplementary material for: Impact of Implementing Alternative Human Papillomavirus (HPV) Vaccination Strategies in Kenya: A Modeling Study
Source: MDM Policy Pract. 2026 Jun 29;11(1):23814683261456695. doi: 10.1177/23814683261456695 (PMC13320054; doi:10.1177/23814683261456695)
Supplement: sj-docx-1-mpp-10.1177_23814683261456695 – Supplemental material for Impact of Implementing Alternative Human Papillomavirus (HPV) Vaccination Strategies in Kenya: A Modeling Study [file sj-docx-1-mpp-10.1177_23814683261456695.docx]

**Appendix S1: Historical HPV vaccination coverage (2019-2023)**

| Year | HPV1 | Hpv2 |
| --- | --- | --- |
| 2019 | 24% | - |
| 2020 | 32% | 16% |
| 2021 | 26% | 9% |
| 2022 | 16% | 15% |
| 2023 | 55% | 44% |

**Appendix S2: Impact of various scale-up options of the current HPV vaccination strategies in Kenya, assuming 30% access to cancer treatment**

| **Implementation scenario** | **Total cases averted** | **Total DALYS averted**  **(Thousands)** | **Total deaths averted** | **Years of life saved**  **(Thousands)** | **Costs (millions, 2024 USD)** | | | |
| --- | --- | --- | --- | --- | --- | --- | --- | --- |
|  |  |  |  |  | **Vaccine** | **Delivery** | **Total vaccination** | **Treatment** |
| Scenario-0 | 172,713 | 2,701.6 | 142,284 | 2,580.9 | 62.7 | 19.0 | 81.7 | 695.3 |
| Scenario-1 | 188,867 | 2,977.6 | 155,302 | 2,845.8 | 77.3 | 21.4 | 98.7 | 687.0 |
| Scenario-2 | 205,251 | 3,257.4 | 168,521 | 3,114.2 | 95.5 | 23.6 | 119.1 | 678.6 |
| Scenario-3 | 204,391 | 3,217.6 | 168,067 | 3,074.9 | 144.7 | 21.4 | 166.1 | 679.1 |
| Scenario-4 | 246,942 | 3,908.6 | 204,837 | 3,735.7 | 99.0 | 29.1 | 128.1 | 657.3 |
| Scenario-5 | 188,867 | 2,977.6 | 155,302 | 2,845.8 | 99.1 | 21.4 | 120.5 | 687.0 |
| Scenario-6 | 174,902 | 2,739.1 | 144,045 | 2,616.9 | 67.0 | 19.3 | 86.3 | 694.2 |
| Scenario-7 | 184,035 | 2,951.3 | 150,689 | 2,823.1 | 58.7 | 18.0 | 76.7 | 689.5 |
| Scenario-8 | 184,035 | 2,951.3 | 150,689 | 2,823.1 | 74.9 | 18.0 | 92.9 | 689.5 |
| Scenario-9 | 210,259 | 3,375.5 | 172,111 | 3,229.1 | 96.8 | 18.0 | 114.8 | 676.0 |
| Scenario-10 | 188,867 | 2,977.6 | 155,302 | 2,845.8 | 49.0 | 21.3 | 70.3 | 687.0 |
| Scenario-11 | 184,035 | 2,951.3 | 150,689 | 2,823.1 | 43.3 | 18.0 | 61.3 | 689.5 |
| Scenario 12 | 173,316 | 2,759.5 | 141,912 | 2,638.7 | 104.9 | 19.6 | 124.5 | 695.0 |
